# Evaluation of Treatment Patterns and Direct Costs Associated with the Management of Neuropathic Pain

**DOI:** 10.1155/2020/9353940

**Published:** 2020-03-30

**Authors:** Manuel E Machado-Duque, Andres Gaviria-Mendoza, Jorge E Machado-Alba, Natalia Castaño

**Affiliations:** ^1^Grupo de Investigación en Farmacoepidemiología y Farmacovigilancia, Universidad Tecnológica de Pereira-Audifarma S.A. Pereira, Pereira, Colombia; ^2^Grupo Biomedicina. Fundación Universitaria Autónoma de Las Américas, Pereira, Colombia; ^3^Pfizer S.A.S, Bogotá, Colombia

## Abstract

**Background:**

Neuropathic pain has a prevalence of 2–17% in the general population. Diagnosis and treatment of neuropathic pain are not fully described in different populations. The aim was to determine the treatment patterns and direct costs of care associated with the management of neuropathic pain from the onset of the first symptom to up to two years after diagnosis.

**Methods:**

From a drug-claim database, a cohort of randomly selected outpatients diagnosed with neuropathic pain was obtained from an insurer in Colombia and followed up for two years after diagnosis. The clinical records were reviewed individually to identify the study variables, including the time needed to make the diagnosis, the medical and paraclinical resources used, the pharmacological therapy for pain management, and the direct costs associated with care.

**Results:**

We identified 624 patients in 49 cities, with a mean age of 50.3 ± 14.1 years, of which 324 were men (51.9%). An average of 90 days passed from the initial consultation until the diagnosis of neuropathic pain, the most frequent being lumbosacral radiculopathy (57.9%). 34.5% of the cohort had at least one diagnostic imaging procedure, and 16% had an electromyography. On average, they were treated by a general practitioner twice. 91.7% received initial treatment with tramadol, carbamazepine, amitriptyline, imipramine, or pregabalin, and 60.4% received combined therapy. The mean cost of care for two years for each patient was US$246.3.

**Conclusions:**

Patients with neuropathic pain in Colombia are being diagnosed late, are using therapeutic agents not recommended as first-line treatment by clinical practice guidelines, and are being treated for short periods of time.

## 1. Introduction

Neuropathic pain has a prevalence of 2 to 17% in the general population according to different studies [1]. The pathologies that generally are accepted to cause neuropathic pain include trigeminal neuralgia, painful diabetic polyneuropathy, and postherpetic neuralgia, among others [2]. Neuropathic pain is characterized by generation of intense suffering for those who experience it and affects the quality of life of these patients and their caregivers [3]. Neuropathic pain is defined as pain produced by an injury or illness that affects the somatosensory system [2–4].

Different therapeutic strategies are available to deal with neuropathic pain, including both pharmacological and nonpharmacological interventions [5, 6]. The pharmacological interventions recommended for neuropathic pain management in clinical practice guidelines have been classified according to their role in the etiology or underlying pathophysiological mechanisms [3, 5, 7]. First-line treatments include tricyclic antidepressants and anticonvulsants, such as pregabalin and gabapentin, which have shown efficacy for pain management [3, 5, 7]. As second- and third-line treatments, lidocaine, other antidepressants, especially those with dual action, other anticonvulsants, and opioids, such as tramadol and oxycodone, have been used, although these drugs carry a risk of long-term abuse [3, 5, 8, 9].

Occasionally, patients are treated with other types of treatments, such as analgesics, which have not proved efficacy for this specific condition. Also, some patients could be receiving suboptimal doses or at inappropriate intervals. Consequently, adherence of these treatments may be poor, which can lead to a low therapeutic response and persistence of symptoms [10, 11].

On the other hand, accurate diagnosis is challenging due to the heterogeneity of positive and negative sensorineural symptoms; thus, developing simple protocols for pain classification and correct treatment sometimes is not plausible, which leads to unnecessary use of health system resources and administration of inappropriate treatments [12, 13]. In the United States, the estimated health system cost for management of chronic pain syndromes exceeds $600 billion dollars per year, greater than the annual costs of heart disease, cancer, and diabetes [14].

In Colombia, the annual cost of chronic pain is unknown. Some statistics indicate that about 35 to 50% of the general population suffers chronic pain, [1] suggesting that there may be a large amount of resources allocated to address this type of ailments. The Health System of Colombia offers two universal coverage regimens: a contributory regimen paid by the employer and the worker and a regimen subsidized by the state. Both have benefit plans that include several of the analgesics necessary to treat pain.

Due to the implications of pain for the quality of life of patients, complexity involved in diagnosing the neuropathic pain, and the potential budget impact to the Health Care System, our objective was to determine the treatment patterns and direct costs for medications, consultations, and diagnostic aids associated with the management of neuropathic pain. We analyzed these factors from the onset of a first diagnosis of any type of pain until the patient was actually diagnosed with neuropathic pain and then during a two year follow-up. Additionally, we analyzed persistence, changes, and combinations used for the treatment of neuropathic pain.

## 2. Methods

This was a longitudinal retrospective study. Patients diagnosed with neuropathic pain who were attended by a health insurer (“health maintenance organization”-HMO) in Colombia between 2002 and 2017 were identified from a drug-claim database. This database includes more than 2 million people from the HMO of this study. Two periods of time were evaluated. The initial period began from the appearance of the first diagnosis of pain for each patient to the time of the diagnosis of neuropathic pain (the index date). The second period comprised the following two years after the diagnosis of neuropathic pain was established. From the patients identified in the drug-claim database, a random sample was calculated to select those who participated in the study.

Subsequently, the drug-claim database, electronic medical records (EMRs) of each selected case, and billing information were used to extract the study variables. Using the EMRs, the clinical course was evaluated from the first diagnosis of pain until the diagnosis of neuropathic pain was made. This evaluation comprised identification of the resources associated with care, including medical appointments with specialists and general practitioners, the medications dispensed, and the laboratory and imaging services provided. The variables were parameterized based on the data in EMRs and the published clinical studies.

During the two-year follow-up period after diagnosis, the treatment patterns were identified, considering the drugs used, their dosages, persistence, or changes in medication, the current cost of the drugs, and the use of health resources. The International Association for the Study of Pain (IASP) diagnostic guidelines were used to evaluate the symptoms of neuropathic pain [15]. Finally, the results were consolidated and validated.

We included patients older than 18 years with complete medical records and the diagnoses associated with neuropathic pain according to the International Classification of Diseases (ICD-10), G54.1, G53, G54, and G56-G62, who were receiving medications approved by the National Institute for the Surveillance of Drugs and Foods of Colombia (Instituto Nacional de Vigilancia de Medicamentos y Alimentos, INVIMA, Spanish abbreviation) for this indication prior to 2015 and who had been diagnosed and treated in the health insurer network. We also included the code for fibromyalgia (M79.7), which is not formally defined as a neuropathic pain, but it is a condition in which many of the medications used to treat neuropathic pain can be useful [16].

Patients with the following diagnoses were excluded: acute and transient psychotic disorders (F23), depressive episode (F32), recurrent depressive disorder (F33), persistent mood (affective) disorders (F34), epilepsy (G40), seizures not classified elsewhere (R56), phobic anxiety disorders (F40), and other anxiety disorders (F41).

A simple random sample of 646 patients was estimated, based on a total of 49,321 patients affiliated with the HMO who had the diagnoses of the study. An expected proportion of 18.9% (according to treatment persistence), accuracy of 3%, and a confidence level of 95% were used.

The following groups of variables were considered:

- Sociodemographic: age, sex, city of residence, and insurance regime
- Clinical: main diagnosis considered in the inclusion criteria, diagnostic images related to the diagnosis of neuropathic pain, specific tests for the diagnosis of neuropathic pain, date of the diagnosis of neuropathic pain, comorbidities, and interventions
- Pharmacological: medications for the management of neuropathic pain, dosages, dates of beginning and end of treatment, route and frequency of administration, cause of suspension of study drugs, proportion of days covered, use of combination therapy, start and end dates for combination therapy and concomitant medications
- Use of resources: costs of study treatment, number of visits to medical specialists, type of medical specialists, type and number of laboratory and imaging services, and appointment costs for medical specialists

The costs of medical care (direct costs of medical consultations, specialties, and subspecialties) and of each paraclinical diagnostic test were evaluated during the second period of the study. The payer's perspective from a microcosting approach was used to determine gross costs using the Colombian pesos and US dollars for quantification, according to the representative exchange rate given by the Central Bank of Colombia, 1 US = COP$2,984.

Medical care and paraclinical costs were assessed using the Colombian Social Security Institute's 2001 fee manual, with an additional 30% added to compensate for 2018 prices, as recommended by the Institute of Health Technology Evaluations of Colombia (Instituto de Evaluaciones de Tecnologías en Salud de Colombia, IETS).

The costs of the drugs were obtained from the invoicing prices of the logistic operator (Audifarma SA) for the corresponding HMO. The price of each medication was considered according to the average cost for the treatment year during the two-year follow-up.

### 2.1. Statistical Analysis

For the data analysis, the statistical package SPSS Statistics, version 24.0 (IBM, USA) for Windows was used. The information was collected by physicians following a data extraction template developed in Microsoft Excel. The template was parameterized according to the possible responses and the availability of information. It was validated in a pilot test of randomly selected medical records with the aim of standardizing criteria and concepts. Frequency and proportion analyses were performed for categorical variables, and measures of central tendency, position, and dispersion were conducted for quantitative variables according to the data normality determined using the Kolmogorov–Smirnov test.

The study was approved by the Bioethics Committee of the Universidad Tecnológica de Pereira (Code : CBE-SYR-162016) under the “no risk” research category. The ethical principles established by the Declaration of Helsinki were respected. In no case was personal data collected for the patients.

## 3. Results

A total of 624 randomly selected patients were evaluated after excluding 22 due to identification of a concomitant diagnosis of anxiety disorder or depressive episodes during the EMR review. The mean age at the beginning of the cohort was 50.3 ± 14.1 years, 324 were men (51.9%), and all patients were followed for 24 months. The patients were from 49 different cities in Colombia.

We found a median of 7 days (interquartile range: 0–82 days) and an average of 90.6 ± 214 days between the pain-related diagnosis and neuropathic pain diagnosis. The most frequent diagnosis was lumbar radiculopathy (more than half of the cases).  Table 1 presents the frequencies of the diagnoses recorded in the medical record and the mean number of days until the diagnosis was made, which varied according to the type of pain identified.

Comorbidities were identified in 367 patients (58.8%). The most frequent comorbidities were arterial hypertension (*n* = 194, 31.1%), dyslipidemia (*n* = 109, 17.5%), diabetes mellitus (*n* = 78, 12.5%), migraine (*n* = 25, 4.0%), obesity (*n* = 23, 3.7%), and peripheral vascular disease (*n* = 21, 3.4%). A total of 236 patients were receiving concomitant medications for any of these conditions, the most common of which were losartan (*n* = 89, 14.2% of patients), atorvastatin (*n* = 82, 13.1%), omeprazole (*n* = 73, 11.3%), enalapril (*n* = 57, 9.1%), acetylsalicylic acid (*n* = 45, 7.2%), gemfibrozil (*n* = 42, 6.7%), insulin (*n* = 41, 6.6%), and metformin (*n* = 40, 6.4%).

Table 2 shows the resources associated with health care attention related to the diagnosis of the neuropathic pain and the invasive procedures used for therapeutic purposes. A total of 56.4% of the patients had at least one diagnostic image, being the most frequent lumbosacral radiography (36.2%). Limb electromyography was reported in 16% of the population, and only 11.4% of patients reported invasive therapeutic procedures such as joint injection, nerve block, and hemilaminectomy.

The main drugs initiated for pain management and those used at any time during follow-up are shown in Table 3. Initial management of the neuropathic pain by the attending physician mainly was comprised of combination therapy (*n* = 377, 60.4%), and the most commonly used therapies were tramadol plus acetaminophen (*n* = 44, 7.1%), carbamazepine plus acetaminophen with codeine (*n* = 24, 3.8%), and amitriptyline plus acetaminophen with codeine (*n* = 32, 3.4%), although 70 other combinations were identified.

**Figure 1:**
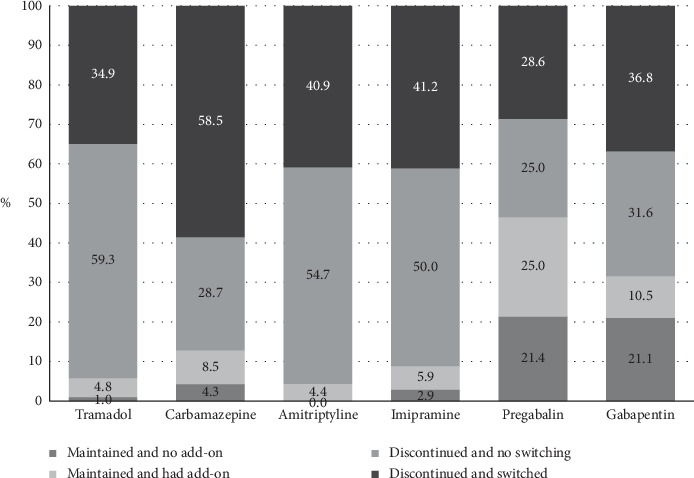
Proportion of persistence and discontinuation of the drugs most used for the treatment of neuropathic pain in Colombia.

Figure 1 shows the changes in the drugs used for the management of neuropathic pain, including the additions and suspensions of drugs during the two years of follow-up. We observed that most medications were discontinued or substituted with other medications, except for pregabalin and gabapentin which reported more frequency of patients without treatment switch (46.4% and 31.6%, respectively).

All patients had an average of two consultations with a general practitioner per year during the follow-up period, with 228 (36.8%) visiting a specialist, particularly a family doctor (*n* = 63, 10.1%), orthopedics (*n* = 60, 9.7%), psychiatry (*n* = 56, 8.9%), occupational medicine (*n* = 55, 8.9%), neurosurgery (*n* = 30, 4.9%), and internal medicine (*n* = 28, 4.5%). A total of 143 (22.9%) patients had more than one specialized consultation.

### 3.1. Costs

Table 4 shows the direct costs used to make the diagnosis with paraclinical support, such as imaging and electromyography, for each type of neuropathic pain included in the study, as well as the average costs associated with the care provided by the attending physicians and the costs of pharmacological therapy per year and during the two years of follow-up. US$13.80 per specialized consultation and US$10.50 per general practitioner were considered. On average, the cost per patient since the pain-related diagnosis and during the two years of follow-up was US$246.30. This cost was equal to the value of the per capita payment recognized by the Colombian Health System to the insurer for each patient affiliated per year (USD 250) at the time of the study.

## 4. Discussion

The present study was able to identify and describe the time and resources required for the diagnosis of a variety of pathologies associated with neuropathic pain and determine the pharmacological treatment patterns used for these patients during a two-year follow-up. We also identified changes and adjustments to such therapy. For the first time, these clinical scenarios are described in Colombia, providing useful and practical information regarding the diagnosis and treatment of neuropathic pain in patients affiliated with a national HMO.

The condition most frequently associated with neuropathic pain was lumbar radiculopathy. The physicians took more than 100 days to establish the diagnosis, which was above the mean of the entire sample evaluated. It was also the most expensive condition due to the radiological resources used. This situation generates warning for clinical staff and decision makers to optimize the allocation of resources for diagnosis, searching time-saving strategies, justifying the use of diagnostic test correctly, or adapting clinical practice guidelines for the management of lumbar radiculopathies. In addition, fibromyalgia required more than 200 days until diagnostic confirmation [8, 10, 17, 18]. No publications were found that measured or determined the time needed to establish the diagnosis of neuropathic pain.

European studies conducted in France, the United Kingdom, and Spain reported that anticonvulsants (pregabalin, gabapentin, and carbamazepine) were the most frequently prescribed initial drugs (68% vs 34.1% in this study), followed by opioids (25% vs 33.5% for tramadol in Colombia) and tricyclic antidepressants (17% vs 27.4% in Colombia) [11, 19]. In a study carried out in the United States, initial use of anticonvulsants was similar to that of European studies, but opioid use was lower (<13%), with a higher proportion of selective or dual-action antidepressants compared to tricyclics. Conversely, in our study, treatment was started with selective antidepressants in only 2.7% of patients and 21.1% received this treatment throughout the follow-up [11].

Tramadol was the most commonly used as initial therapeutic analgesic, and one-third of the patients were receiving it. However, a high proportion underwent a suspension or change to another medication. Opioids are used for the management of low back pain with radiculopathy only in acute episodes according to current recommendations, although studies have supported their effectiveness in reducing pain and improving activity, especially for peripheral neuropathic pain [3, 8, 20]. However, their long-term management is not recommended due to the risks of dependence and abuse, which may explain why this sample of patients only received these drugs during the initial period [21]. The average dose used was much lower than that recommended according to the parameters of the Defined Daily Dose (DDD) comparison, which might be due to different factors, such as the pharmaceutical presentation used (especially in drops) or the lack of clear recommendations for their use in radiculopathy.

Carbamazepine, amitriptyline, and imipramine accounted for approximately half of the prescriptions used as initial therapy; these have some evidence of their effectiveness in the treatment of long-term neuropathic pain. Nevertheless, a significant proportion of treatment discontinuation was found during the follow-up, with persistence of use in approximately 10% of cases. This situation may be associated with poor tolerability due to the appearance of adverse effects or therapeutic failure [3, 10, 20, 22–24]; at the same time, this group of drugs has shown less persistence of use over time [11].

Pregabalin and gabapentin were prescribed with low frequencies as an initial therapy (4.5 and 3.3%, respectively). However, their use increased throughout the follow-up period. These drugs were used in 34.2% of the patients at any time during follow-up and were the group of drugs that were most often used at the end of the study period (the last month). The persistence was also high for these drugs, with almost half of the patients who started with them continuing the therapy, alone or combined with another drug, after the first three months; in contrast, the persistence of use of all of the other analgesics was only approximately 10%. These results are similar to those of a study conducted in the United States, which found that these two drugs showed the greatest persistence [11].

The predominance in the use of pregabalin in the male population is striking; previous reports have shown that women with ages similar to those reported in this study use it more frequently [25]. The average dose used was close to 150 mg/day, which could be considered insufficient because different studies reported that doses greater than 300 mg/day were more effective for pain control than lower doses [26, 27]. In addition, a lack of dose increases in the usual management of these patients has been previously reported, possibly due to difficulties associated with poor tolerability or explained by proper prescription behaviors of the treating physicians. We were unable to rule out that the patients were in a gradual process of increasing the dose to reach optimal management [11, 28].

During the observation time were frequent the combinations in the treatment, with periods of acute and chronic use or changes and moments without therapy. This finding might suggest some type of difficulty in the management of this group of pathologies, in which drugs not approved for these indications were being chosen and prescribed at suboptimal doses or with different efficacy/tolerability ratios [11]. In addition, the vast majority of patients did not continue pharmacological treatment during the follow-up period and received management for brief periods that might have coincided with exacerbations of the pathology. This result may suggest that the patients are not being effectively treated, which may affect their quality of life and be associated with complications [2, 3].

The cost of pharmacological therapy was approximately $62 per year, particularly for generic drugs that generally had lower prices. The lowest cost therapies were those that included commonly used analgesics, such as acetaminophen or different nonsteroidal anti-inflammatories, whereas opioids, such as codeine or hydrocodone, and anticonvulsant drugs had higher costs [29, 30].

This study has some limitations as a descriptive study, such as the inclusion of patients diagnosed with neuropathic pain from a single HMO in the country and the exclusion of those who have not been diagnosed or are not being treated pharmacologically. Also, we could not evaluate the medical records of the consultations of some patients attended by a group of specialist doctors who were outside the HMO's network and could not establish the use of resources and the costs associated with emergency care and during hospitalization. Finally, the calculated costs are a general estimate at the current prices, but each service provider may have different rates established for interventions, consultations, and diagnostic tests. Thus, conclusions should be applied to populations with similar demographic and insurance characteristics.

Multiple strengths are also recognized, such as the size of the sample for an observational study of this type, the rigor of data collection, the different sources of information for monitoring the medicines used, and the associated costs based on a population of more than 2 million insured patients.

## 5. Conclusions

With the above findings, this study concludes that patients with neuropathic pain are being diagnosed on average 90 days after their initial consultation, using significant imaging resources, general medical care, and different specialties. Additionally, these patients are initially treated with drugs that are not considered first-line for this type of pain according to clinical practice guidelines and are prescribed at doses lower than recommended for relatively short periods, with multiple suspensions and substitutions with new drugs over a two-year follow-up [23].

These results suggest that making the diagnosis and undertaking and maintaining adequate therapy is difficult, and thus, an effort needs to be made to ensure that those responsible for healthcare take measures to guarantee the opportunity and quality of the service. Studies must be carried out to establish the effectiveness of neuropathic pain treatments and develop clinical practice guidelines that seek to improve diagnostic processes and provide adequate, effective, and safe treatment in these patients.

## Figures and Tables

**Table 1:** Main diagnoses and time until the diagnosis of neuropathic pain in 624 patients, Colombia.

Diagnosis	Frequency (%) *n* = 624	Mean time until neuropathic pain diagnosis (days)
ICD10 diagnosis		
Lumbosacral neuritis or radiculitis	361 (57.9)	103.3 ± 239.6
Other mononeuropathies	101 (16.2)	48.4 ± 159.5
Mononeuropathies of upper limb	64 (10.3)	58.7 ± 138.5
Other polyneuropathies	33 (5.3)	123.1 ± 172.4
Mononeuropathies of lower limb	20 (3.2)	72.7 ± 172.5
Diabetic polyneuropathy	18 (2.9)	97.1 ± 299.9
Fibromyalgia	11 (1.8)	209.7 ± 215.8
Diabetic mononeuropathy	9 (1.4)	15.7 ± NA
Idiopathic neuropathy	4 (0.6)	27.5 ± NA
Inflammatory polyneuropathy	2 (0.3)	16.5 ± NA
Mononeuropathy in diseases classified elsewhere	1 (0.2)	8 ± NA

Clinical record diagnosis		
Lumbago with sciatica	196 (31.4)	
Neuralgia and neuritis, unspecified	107 (17.1)	
Lumbar and other intervertebral disc disorders with radiculopathy	62 (9.9)	
Lumbago, unspecified	25 (4.0)	
Radiculopathy	20 (3.2)	
Cervicalgia	17 (2.7)	
Zoster without complication	13 (2.1)	
Carpal tunnel syndrome	13 (2.1)	
Other diagnoses	171 (27.5)	

ICD10 : International Classification of Diseases, 10th revision. NA: not applicable.

**Table 2:** Diagnostic tests and therapeutic procedures used patients with neuropathic pain, Colombia.

Tests and procedures	Frequency	%
Number of diagnostic images per patient		
0	283	45.4
1	215	34.5
2	112	17.9
3	14	2.2

Diagnostic images (more frequent)		
Lumbosacral radiography	226	36.2
Lumbosacral MRI	53	8.5
Spine MRI	37	5.9
Cervical, thoracic, lumbar or sacrum segments CT scans	35	5.6
Cervical radiography	27	4.3
Dorso-lumbar radiography	13	2.1
Cervical MRI	12	1.9
Hip and coxofemoral joint radiography	8	1.3

Other diagnostic tests		
Limb electromyography	100	16.0

Invasive therapeutic procedures		
Number of patients with interventions	71	11.4
Joint injection	35	5.6
Nerve block (facet, medular, peripheral)	15	2.5
Hemilaminectomy	10	1.6

MRI: magnetic resonance imaging; CT: computed tomography.

**Table 3:** Patterns of use of initial medications prescribed for the treatment of patients with neuropathic pain in Colombia.

Medication	At start^a^		Any time^b^		Mean dose (mg/day)	Most frequent interval	nDDD	Mean age ± SD
	*n*	%	*n*	%				
Tramadol	209	33.5	411	65.9	22	3	0.07	47.0 ± 13.0
Carbamazepine	164	26.3	221	35.4	234.1	1	0.23	52.0 ± 14.2
Amitriptyline	137	22	240	38.5	26.3	1	0.35	48.5 ± 14.4
Imipramine	34	5.4	74	11.9	18.5	1	0.18	50.8 ± 12.2
Pregabalin	28	4.5	149	23.9	171.4	1	0.57	64.9 ± 12.9
Gabapentin	19	3	64	10.3	357.8	1	0.20	51.1 ± 14.5
Lidocaine	14	2.2	38	6.1	–––	3	–––	60.8 ± 13.4
Fluoxetine	13	2.1	92	14.7	21.5	1	1.05	52.9 ± 8.5
Sertraline	4	0.6	40	6.4	50	1	1.00	51.7 ± 14.9
Valproic acid	2	0.3	31	5.0	500	1 and 3	0.33	49.0 ± 6.3

nDDD: ratio between the Defined Daily Dose (DDD) and the dose used; SD: standard deviation.^a^ Frequency of use of each medication at the beginning of the follow-up.^b^ Frequency of use of each medication during any time of follow-up.

**Table 4:** Direct costs associated with diagnosis, medications, and follow-up medical consultations for patients with neuropathic pain in Colombia.

Direct costs	Cost per patient US dollars (mean)	Total cost US dollars
Neuropathic pain diagnosis		
*Diagnostic images (n* *=* *624)– mean cost*^*∗*^	$ 60.9 ± 123.0	
*Lumbosacral neuritis or radiculitis (n* *=* *361)*	$ 88.3 ± 145.3	$ 31,883.1
*Other mononeuropathies (n* *=* *101)*	$ 15.7 ± 61.2	$ 1,590.3
*Mononeuropathies of upper limb (n* *=* *64)*	$ 29.0 ± 75.4	$ 1,855.7
*Other polyneuropathies (n* *=* *33)*	$ 38.3 ± 91.4	$ 1,264.7
*Mononeuropathies of lower limb (n* *=* *20)*	$ 17.7 ± 51.9	$ 354.8
*Diabetic polyneuropathy (n* *=* *18)*	$ 7.0 ± 17.8	$ 126.1
*Diagnostic Imaging (n* *=* *341)*^*∗∗∗*^	$ 111.4 ± 123.3	$ 37,991.8
*Electromyography and nerve conduction (n* *=* *100)*^*∗∗∗*^	$ 35.1 ± 13.4	$ 3,510.0
*Average total cost (n* *=* *624)*^*∗*^	$ 66.6 ± 125.5	$ 41.501.9

Medications (during the two years of follow-up)		
*Medication cost per year - mean (n* *=* *624)*^*∗*^	$ 61.5 ± 47.5	$ 38,364.3
*Total Cost of medicines - (2 years) (n* *=* *624)*^*∗*^		$ 76,728.5

Medical consultations (during the two years of follow-up)		
*Mean cost of medical consultation (general and specialties) (n* *=* *624)*^*∗*^	$ 62.2 ± 27.0	$ 38,809.5

^*∗*^mean considering the total population. ^*∗∗∗*^mean considering only those who used the test.

## Data Availability

The data used to support the findings of this study are available from the corresponding author upon request.
